# Anonymity preserving and round effective three-party authentication key exchange protocol based on chaotic maps

**DOI:** 10.1371/journal.pone.0213976

**Published:** 2019-03-20

**Authors:** Kyongsok Pak, Songho Pak, Cholman Ho, Myongsuk Pak, Choljin Hwang

**Affiliations:** College of Information Science, Kim Il Sung University, Pyongyang, DPR of Korea; King Saud University, SAUDI ARABIA

## Abstract

Three-party authentication key exchange (3PAKE) is a protocol that allows two users to set up a common session key with the help of a trusted remote server, which is effective for secret communication between clients in a large-scale network environment. Since chaotic maps have superior characteristics, researchers have recently presented some of the studies that apply it to authentication key exchange and cryptography. Providing user anonymity in the authentication key exchange is one of the important security requirements to protect users' personal secrets. We analyse Lu et al.'s scheme which attempts to provide user anonymity and we prove that his scheme has errors in the key exchange phase and password change phase. We propose a round-effective three-party authentication key exchange (3PAKE) protocol that provides user anonymity and we analyse its security properties based on BAN logic and AVISPA tool.

## 1. Introduction

Along with the rapid development of the information technology and computer network, user authentication plays an important role in protecting resources, service and user’s personal information in the computer network. The authentication key exchange protocol is one of the important mechanisms of network security aimed at setting a session key for secret communication between users via an open network. The authentication key exchange protocol is keys exchange for the secret communication based on authentication between the communicating parties in essence. The authentication key exchange protocol can be classified into Two-Party Authentication Key Exchange (2PAKE), Three-Party Authentication Key Exchange (3PAKE), and Multi-Party Authentication Key Exchange (MPAKE) depending on the number of participating in the key exchange. The key point of the 3PAKE protocol is that it does not need to remember various passwords for each user, and can establish secret communication between users with the help of a trusted remote server.

### 1.1 Cryptography for key exchange

Since the authentication key exchange protocol was proposed by Bellovin and Merritt [1] in 1992, there have been many studies on 2PAKE protocol [2,3], 3PAKE protocol and MPAKE[4–6] protocol based on the various cryptography algorithms for decades. The researchers used the Diffie-Hellman (DH) key exchange scheme [7–18], the Elliptic Curve Cryptosystem (ECC) based key exchange scheme [19–26], and the Chebyshev chaotic maps based key exchange scheme [27–38] for key exchange in 3PAKE protocol. The DH key exchange scheme based on modular exponentiation [39] requires a lot of computational cost. The ECC based scheme [40], in which the key length is small and the computational cost is low, has been used for key exchange. The ECC based scheme is more efficient in terms of key length and computational cost than the DH key exchange scheme using modular exponentiation [41].

In 2008, in order to enhance the property of the Chebyshev chaotic maps, Zhang [42] proved that the semi-group property holds for Chebyshev polynomials [43] defined over the interval (−∞, +∞), and Chebyshev chaotic maps based key exchange schemes were widely used in the 3PAKE protocol. Chebyshev chaotic maps based scheme has advantages such as high safety, low computational cost, simple encryption, small storage capacity requirement, and low bandwidth [37, 44, 45]. Therefore, compared to DH and ECC based scheme, Chebyshev chaotic maps based scheme is more suitable for the wireless sensor network and the authentication system using smart card. In 2016, Kumari et al.[46] proposed mutual authentication and key agreement scheme for wireless sensor networks using Chebyshev chaotic maps, in which they described different chaotic maps that could be used in digital authentication and discussed a design methodology to present a robust authentication and key agreement for wireless sensor networks, and proposed a new authentication scheme for wireless sensor networks which provides user anonymity. However, his scheme is vulnerable to session-specific temporary information attack, sensor node impersonation attack, man-in-the-middle attack [47].

### 1.2 User authentication schemes in 3PAKE

In 3PAKE, the authentication server authenticates users and exchanges session key between users. In order for server to authenticate users in the 3PAKE protocol, researchers applied user password scheme [7–15, 19, 20, 27, 48], a combination of server public key and user password [17, 18, 23–26, 30–36], shared secret key scheme [21, 22, 28, 29, 49–51], and a combination of shared secret key and server public key [16, 38, 52–54].

The user password scheme without public key and shared secret key is easily revealed by password guessing attack as the information entropy of the password is low [8]. For example, in 2009 Huang [7] designed a 3PAKE protocol based on user password. However, Yoon et al. [10] proved that Huang’s scheme is vulnerable to off-line password guessing attack and undetectable on-line password guessing attack. Wu et al. [17] proved that Huang’s scheme is vulnerable to key-compromise impersonate attack, and proposed an updated 3PAKE protocol using user password and server public key. On the other hand, Chang et al. [8] proposed efficient 3PAKE protocol based on user password using modular exponentiation, and Wu et al. [19] pointed out that his scheme is vulnerable to password guessing attack and designed a 3PAKE protocol based on user password, however Wu et al.’s scheme is vulnerable to key-compromise impersonate attack [18]. Tso [12] also pointed out that Chang et al.’s scheme is vulnerable to password guessing attack, and Tso’s scheme is vulnerable to the off-line password guessing attack and the impersonate attack [14]. Youn et al. [13] also designed efficient 3PAKE protocol based on user password, but his scheme is vulnerable to impersonate attack [15]. Farash et al. [27] proposed 3PAKE protocol based on the user password and the chaotic maps, but Li et al. [38] pointed out that his scheme is vulnerable to password disclosure attack, user impersonate attack, and off-line password guessing attack, and proposed a 3PAKE protocol based on chaotic maps with shared secret key.

The server public key scheme has to construct key management mechanism, so the protocol design is relatively complex and computational complexity is increased. But, using this scheme in the 3PAKE can provide user anonymity by encrypting the message exchanged between the user and the server. In 2014, Xie et al. [23] proposed a 3PAKE protocol based on ECC and the server public key, which provides user anonymity. However, his scheme is vulnerable to privileged insider attack, because there is a table stored user's password in the server side. Lou and Huang[24] also proposed a 3PAKE protocol based on ECC and the server public key, in which there is no encryption message using the server public key, but his scheme is vulnerable to off-line password guessing attack and key-compromise impersonate attack [26]. In 2013, Xie et al. [30] and Lee et al. [32] proposed a 3PAKE protocol based on the chaotic map and the server public key. However, Lee et al. [28] pointed out that Xie et al.’s scheme fails to provide user anonymity, is vulnerable to off-line password guessing attack, and has problems with password table management. Hu et al. [34] pointed out that Lee et al.'s scheme does not provide user anonymity and is vulnerable to MITM attack, and Farash et al. [33] pointed out that Lee et al.'s scheme is vulnerable to modification attack and impersonate attack.

In the shared secret key scheme, the server authenticates users by sharing his secret key with them. This scheme is safer than the password based scheme, because there is no user's private information in the server side. For example, it is resistant to privileged insider attack and stolen verifier attack. Tan [21] proposed a 3PAKE protocol based on ECC and the shared secret key, in which user keeps a private key combining with server secret key and user's identification. However his scheme is vulnerable to key-compromise impersonate attack [22]. Li [29] and Islam[50] proposed a 3PAKE protocol based on the chaotic map and the shared secret key, in which user encrypts the data for authentication with his private key derived by the server's private key, but user's identifier is exposed in the message, so their protocol does not provide user anonymity.

Meanwhile, in order to improve the effectiveness and safety of the authentication, there have been studies to implement the 3PAKE protocol by using devices such as smart cards [48–54]. In an authentication key exchange using a password that does not use a public key or shared secret key scheme, the user simply needs to remember the password. However, in an authentication key exchange that uses a public key or shared secret key scheme, the user must have a storage location for storing the server's shared secret key or his public key. The use of smart card not only allows users to carry their own authentication information, but also has the advantage of accessing service by using smart card reading devices anywhere. But in this scheme, there is a risk of losing the smart card. In 2012, Lai et al. [53] proposed the implementation of the 3PAKE protocol to use smart card based on chaotic maps. However, Zhao et al. [52] pointed out that Lai’s scheme is vulnerable to privileged insider attack and off-line password guessing attack, and proposed an updated scheme to use smart card with server public key and shared secret key. Yang et al. [51] proposed a 3PAKE protocol that uses smart card with shared secret key, but Amin et al. [49] proved that Yang’s scheme is vulnerable to off-line password attack, many logged-in user attack, privileged insider attack and has a security weakness in the password change phase, and proposed an updated scheme. In 2015, Xie et al. [48] proposed a 3PAKE protocol that uses smart card based on chaotic maps with user password, but his scheme had several weaknesses. In 2016, Lu et al. [31] pointed out that Xie’s scheme is vulnerable to off-line password attack, user impersonate attack, does not provide user anonymity, and is deficient in session key security. He proposed an updated 3PAKE protocol that provides user anonymity using server public key and user password. However, Lu et al.’s scheme still has a series of weaknesses.

### 1.3 Our contribution

The user’s identifier is a very important personal secret. If user anonymity is not provided, the attacker will know who is currently in the network conversation, and will be able to track the user’s subscription history and current location. Chebyshev chaotic maps based authentication and key exchange scheme is suitable for the authentication system using smart card or the wireless sensor network, which requires low computational cost, simple encryption, small memory size, and low bandwidth. Based on such studies, we analyse the Lu et al.’s scheme [31] and point out its weakness, and propose a round-effective 3PAKE protocol based on chaotic maps using smart cards to provide user anonymity and protect against various attacks. In the proposed scheme, in order to provide the user anonymity the messages exchanged between the sender and the receiver is encrypted with the shared secret key based on the server’s public key, and in order to authenticate the message, we use the user’s private key derived by user’s identifier and the server’s secret key.

In Section 2, we describe the theory of chaotic maps, one-way function and Bio-hashing function, and In Section 3 we review Lu et al.’s scheme. Section 4 presents the proposed scheme, and Section 5 describes the security analysis of the proposed scheme. And Section 6 compares the proposed scheme with the previous schemes in terms of performance.

## 2. Preliminaries

This section describes Chebyshev chaotic maps and their computational problems, and Bio-hashing functions.

### 2.1 Chebyshev polynomials

Chebyshev polynomial *T*_*n*_(*x*) is defined as follows[43].

*T*_*n*_(*x*) = *cos*(*n·arcos*(*x*)), *x*∈[–1,1], *n*∈*N*

Chebyshev polynomials satisfy the following recursive relationship[43].

*T*_*n*_(*x*) = 2*x*·*T*_*n-*1_(*x*)–*T*_*n-*2_(*x*) (*n*>2),

*T*_0_(*x*) = 1, *T*_1_(*x*) = *x*

### 2.2 The property of Chebyshev polynomials

Chebyshev polynomials have the following two properties[43, 46].

Chaotic property: When *n*>1, Chebyshev polynomial map *T*_*n*_(*x*):[–1,1]→[–1,1] of degree n is a chaotic map with its invariant density $f^{*}(x)=\frac{1}{\pi \sqrt{1-x^{2}}}$, for positive Lyapunov exponent *ln*(*n*) > 0.

Semi-group property: For *r*,*s*∈*N* and any *x*∈[–1,1],*T*_*r*_(*T*_*s*_(*x*)) = *T*_*rs*_(*x*) = *T*_*s*_(*T*_*r*_(*x*)).

### 2.3 Enhanced Chebyshev polynomials

The semi-group property holds for Chebyshev polynomials on the interval (-∞,+∞), which can enhance the property as follows [42, 43]:

*T*_*n*_(*x*) = 2*x*·*T*_*n-*1_(*x*)–*T*_*n-*2_(*x*) *mod p*(*n* ≥ 2, *x*∈(-∞,+∞), *p* is a large prime number),

*T*_*r*_(*T*_*s*_(*x*)) ≡ *T*_*rs*_(*x*) ≡ *T*_*s*_(*T*_*r*_(*x*)) *mod p* (*r*,*s*∈*N*).

### 2.4 Computational problems based on Chebyshev polynomials

CDLP(Chaotic map-based Discrete Logarithm problem): For given two real numbers *x* and *y*, it is infeasible to find the integer *r* by any polynomial time bounded algorithm, where *y* = *T*_*r*_(*x*) *mod p* [28, 42, 43].

CDHP(Chaotic map-based Diffie-Hellman problem): For given three elements *x*, *T*_*r*_(*x*) *mod p* and *T*_*s*_(*x*) *mod p*, it is infeasible to compute the value *T*_*rs*_(*x*) *mod p* by any polynomial time bounded algorithm [28, 42, 43].

### 2.5 Bio-hashing function

The biometric technique is very important for user authentication in the authentication system. Generally, imprint biometric characteristics (face, fingerprint, palm-print etc.) may not be exactly same at each time [49]. To solve this problem, Jina et al. [55] and Lumini et al. [56] proposed and updated Bio-hashing, which was used in many authentication schemes [45, 49, 57, 58]. Bio-hashing is used to map a user's biometric features to a user-specific random vectors [45, 57] and is useful for user authentication mechanisms that use small devices such as mobile devices, smart cards, and so on [57].

## 3. Review of Lu et al.’s scheme

This section shows that the scheme proposed by Lu et al. has series of deficiencies in the design. Lu et al. designed 3PAKE protocol based on chaotic maps providing user anonymity. However, his scheme has some errors in the session key exchange phase and the password change phase. Below is a brief description of the scheme proposed by Lu et al. and its deficiencies.

### 3.1 Lu et al.’s scheme

#### Notations used in his paper

*S*: a remote server.
*A* and *B*: two users.
*ID*_*A*_ and *ID*_*B*_: users’ identities of *A* and *B*.
*pwd*_*A*_ and *pwd*_*B*_: users’ passwords of *A* and *B*.
*k* and *T*_*k*_(*x*) *mod p*: private and public keys of *S*.
*s*: a secret key of *S*.
*q*: shared secret key between *A* and *S*.
*h*_1_(): a one-way hash function.
*h*(): a chaotic maps-based one-way hash function.
*p*: a large prime number.

#### System initialization

The server selects random number *x* ∈ *Z*_*p*_ and private key *k* ∈ [1, *p*+1], computes public key *T*_*k*_(*x*) *mod p* and publishes {*p*, *x*, *T*_*k*_(*x*) *mod p*, *h*(∙)}.

#### Registration

- User *A* submits {*ID*_*A*_, *g*_*A*_ = *h*_1_(*pwd*_*A*_, *r*_*A*_) } to *S*, where *r*_*A*_ is random number.
- Upon receiving the registration request, *S* computes *VPW*_*A*_ = *h*_1_(*ID*_*A*_, *k*)⊕*g*_*A*_. Next *S* randomly chooses a secret key *q* for *A* and sends it to *A* via the secure channel. Note that *q* is kept securely by *A* and is different for each user *A*. Finally, *S* stores *k*⊕*q* and *VPW*_*A*_ into its memory.

#### Session key exchange

Step 1: Using the stored shared secret key *q*, user *A* computes his own version of *C*_*A*_ = *E*_*KAS*_(*ID*_*A*_, *ID*_*B*_, *T*_*a*_(*x*), *F*_*A*_) and sends them to *S*, where *K*_*AS*_ = *T*_*q*_(*T*_*k*_(*x*)), *F*_*A*_ = *h*(*ID*_*A*_, *ID*_*B*_, *T*_*a*_(*x*), *g*_*A*_), *a* ∈ [1, *p*+1] is a random number.
Step 2: Once receiving the message, *S* first derives *q* by computing *k*⊕*q*⊕*k* and derives {*ID*_*A*_, *ID*_*B*_, *T*_*a*_(*x*), *F*_*A*_} by decrypting *C*_*A*_ with computed symmetric key *K*_*AS*_ = *T*_*k*_(*T*_*q*_(*x*)). The next steps are omitted here.

#### Password update

Step 1: *A* selects a new password *pwd*_A_* and computes *R*_*A*_ = *E*_*Tq*(*x*)_(*ID*_*A*_, *h*(*pwd*_A_*, *r*_*A*_), *h*(*pwd*_*A*_, *r*_*A*_), *Z*_*AS*_), *Z*_*AS*_ = *h*(*ID*_*A*_, *T*_*S*1_(*x*), *K*_*AS*_) and sends them to *S*.
Step 2: *S* decrypts *R*_*A*_ to retrieve {*ID*_*A*_, *h*(*pwd*_A_*, *r*_*A*_), *h*(*pwd*_*A*_, *r*_*A*_), *Z*_*AS*_} using the shared secret key *q*. The next steps are omitted here.

### 3.2 Defects in the design of Lu et al.’s scheme

#### Session key exchange

In the registration phase, Lu et al. pointed that *q* is kept securely by *A* and is different for each user *A*, and *S* stores *k*⊕*q* into its memory. Therefore, *S* must keep *k*⊕*q* for each user and can obtain it by user identifier. In the step2 of session key exchange phase, Lu et al. pointed that *S* derives *q* by computing *k*⊕*q*⊕*k* and derives {*ID*_*A*_, *ID*_*B*_, *T*_*a*_(*x*), *F*_*A*_} by decrypting *C*_*A*_ with computed symmetric key *K*_*AS*_ = *T*_*k*_(*T*_*q*_(*x*)). In order for *S* to retrieve *k*⊕*q* of *A*, the *A*’s identifier must be present, but *A*’*s* message *C*_*A*_ is encrypted for providing user anonymity and has not yet been decrypted. Therefore, *S* cannot know user *A*’s identifier, and cannot compute *q* = (*k*⊕*q*)⊕*k*. If *S* stores a single *k*⊕*q* for all users, *S* can decrypt the *A*’s message *C*_*A*_ as in the protocol. But, in this case, other users can also decrypt *A*’s message because they also have *q*, so user anonymity cannot be provided in his scheme.

#### Password update

In the password change step, the same defects exist as seen in the session key exchange step. That is, *S* does not obtain the key *K*_*SA*_ = *T*_*k*_(*T*_*q*_(*x*)) to decrypt the message *R*_*A*_ or cannot update password.

## 4. Proposed scheme

This section describes an improved 3PAKE protocol using smart card that overcomes the limitations of the Lu et al.'s scheme. The proposed scheme consists of four steps: system initialization phase, registration phase, authentication and session key exchange phase, and password change phase. The notation presented in Table 1 is used to describe the proposed schemes in this paper.

**Table 1:** Notation used in proposed scheme.

Notation	Description
IDS	Identifier of trusted server S
SCA, SCB	smart card of user A and B
IDA, IDB	Identifier of user A and B
pwA, pwB	Password of A and B
bmA, bmB	Biometrics of A and B
s	Private key of S
p	A large prime number chosen by S
x	seed of Chebyshev polynomials. x ∈ Zp
Tn(x)	Chebyshev polynomials of degree n
KS	S’s public-key (KS = Ts(x))
H(∙)	One-way hash function (0,1)* → (0, 1)n
h(∙)	Bio-hashing function
EK(∙)	Symmetric encrypt algorithm with secret key K
DK(∙)	Symmetric decrypt algorithm with secret key K
||	String concatenation operator
⊕	XOR operator

### 4.1 System initialization phase

1. *S* selects a large prime number *p* and *x* ∈ *Z*_*p*_ for Chebyshev polynomials *T*_*n*_(*x*).
2. *S* selects secure one-way hash function *H*(∙) and a symmetric encryption/decryption algorithm *E*_*K*_(∙)/*D*_*K*_(∙).
3. *S* selects *s* ∈ [1, *p*+1] and keeps it as his secret key, and then computes public-key *K*_*S*_ = *T*_*s*_(*x*) *mod p*.
4. *S* publishes {*p*, *x*, *K*_*S*_, *H*(∙), *E*_*K*_(∙), *D*_*K*_(∙)} as system’s parameters.

### 4.2 User registration phase

All users who want to exchange session keys using the proposed scheme must register on S.

**Fig 1:**
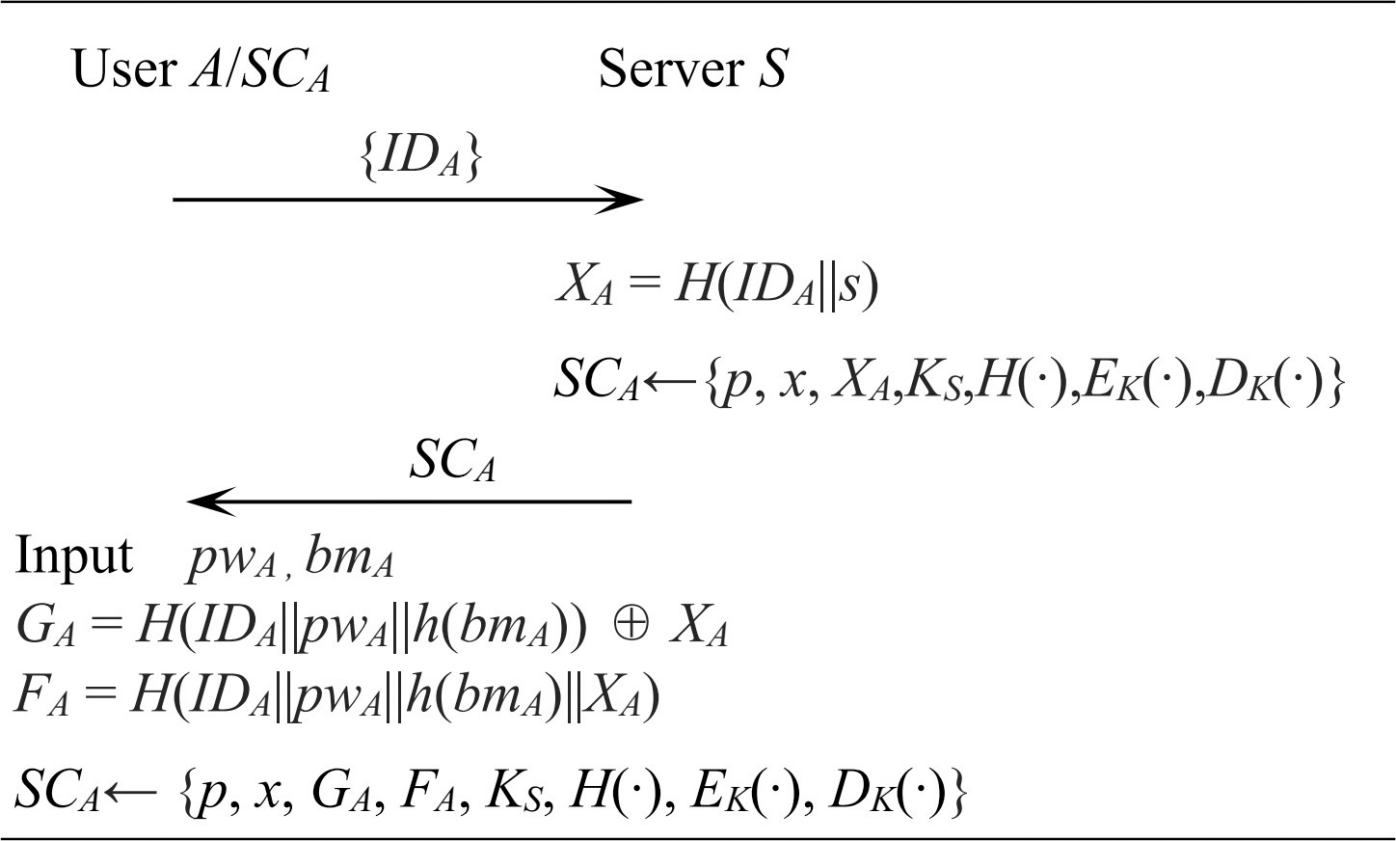
User registration phase of the proposed scheme.

Fig 1 shows an example of user A's registration process.

User *A* sends his/her identifier *ID*_*A*_ to *S* via secure channel. *S* checks whether user *A* has already been registered, otherwise it computes *X*_*A*_ = *H*(*ID*_*A*_||*s*) and stores {*p*, *x*, *X*_*A*_, *K*_*S*_, *H*(∙), *E*_*K*_(∙), *D*_*K*_(∙)} in *SC*_*A*_ and delivers it to user *A* via secure channel.

User *A*, which receives *SC*_*A*_ from *S*, inputs password *pw*_*A*_ and biometric *bm*_*A*_ to access *SC*_*A*_. The *SC*_*A*_ that receives the user input computes *G*_*A*_ = *H*(*ID*_*A*_||*pw*_*A*_||*h*(*bm*_*A*_)) ⊕ *X*_*A*_, *F*_*A*_ = *H*(*ID*_*A*_||*pw*_*A*_|| *h*(*bm*_*A*_)||*X*_*A*_) and stores {*p*, *x*, *G*_*A*_, *F*_*A*_, *K*_*S*_, *H*(∙), *E*_*K*_(∙), *D*_*K*_(∙)} in his memory.

### 4.3 Authentication and session key exchange phase

**Fig 2:**
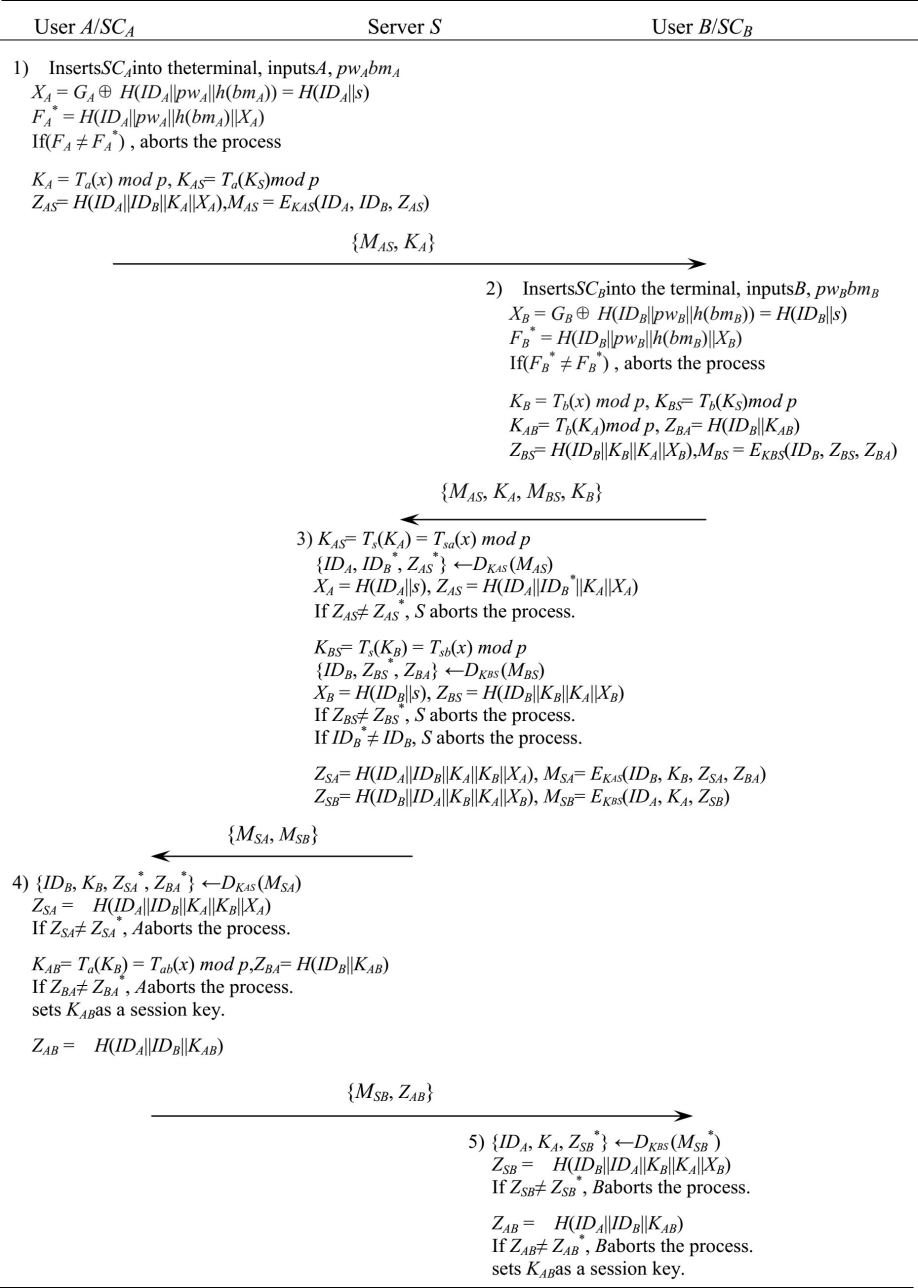
Authentication and session key exchange phase of the proposed scheme.

Fig 2 show the authentication and session key exchange steps of the proposed scheme.

1. User *A* connects his smart card *SC*_*A*_ to the terminal and inputs his identifier *ID*_*A*_, password and biometrics *bm*_*A*_. *SC*_*A*_ computes
  *X*_A_* = *G*_*A*_ ⊕ *H*(*ID*_*A*_||*pw*_*A*_||*h*(*bm*_*A*_)), *F*_A_* = *H*(*ID*_*A*_||*pw*_*A*_||*h*(*bm*_*A*_)||*X*_A_*).
  If *F*_*A*_ ≠ *F*_A_^*^, *SC*_*A*_ aborts the process. Otherwise *SC*_*A*_ selects any *a*∈ [1, *p*+1] and computes
  *K*_*A*_ = *T*_*a*_(*x*) *mod p*, *K*_*AS*_ = *T*_*a*_(*K*_*S*_) = *T*_*as*_(*x*) *mod p*, *Z*_*AS*_ = *H*(*ID*_*A*_||*ID*_*B*_||*K*_*A*_ ||*X*_*A*_), *M*_*AS*_ = *E*_*KAS*_(*ID*_*A*_, *ID*_*B*_, *Z*_*AS*_).
  *A* sends *M*_1_ = {*M*_*AS*_, *K*_*A*_} to *B*.
2. After receiving {*M*_*AS*_, *K*_*A*_} from *A*, *B* connects his smart card *SC*_*B*_ to the terminal and inputs his identifier *ID*_*B*_, password and biometrics *pw*_*B*_. *SC*_*B*_ computes
  *X*_B_* = *G*_*B*_ ⊕*H*(*ID*_*B*_||*pw*_*B*_||*h*(*bm*_*B*_)), *F*_B_* = *H*(*ID*_*B*_||*pw*_*B*_||*h*(*bm*_*B*_)||*X*_B_*).
  If *F*_*B*_ ≠ *F*_B_*, *SC*_*B*_ aborts the process. Otherwise *SC*_*B*_ selects any *b*∈ [1, *p*+1] and computes
  *K*_*B*_ = *T*_*b*_(*x*) *mod p*, *K*_*BS*_ = *T*_*b*_(*K*_*S*_) = *T*_*bs*_(*x*) *mod p*, *K*_*AB*_ = *T*_*b*_(*K*_*A*_) = *T*_*ba*_(*x*) *mod p*,
  *Z*_*BA*_ = *H*(*ID*_*B*_||*K*_*AB*_), *Z*_*BS*_ = *H*(*ID*_*B*_||*K*_*B*_ ||*K*_*A*_||*X*_*B*_), *M*_*BS*_ = *E*_*KBS*_(*ID*_*B*_, *Z*_*BS*_, *Z*_*BA*_).
  *B* sends *M*_2_ = {*M*_*AS*_, *K*_*A*_, *M*_*BS*_, *K*_*B*_} to *S*.
3. After receiving {*M*_*AS*_, *K*_*A*_, *M*_*BS*_, *K*_*B*_} from *B*, *S* computes
  *K*_*AS*_ = *T*_*s*_(*K*_*A*_) = *T*_*sa*_(*x*) *mod p*, {*ID*_*A*_, *ID*_B_*, *Z*_AS_*} = *D*_*KAS*_ (*M*_*AS*_), *X*_*A*_ = *H*(*ID*_*A*_||*s*), *Z*_*AS*_ = *H*(*ID*_*A*_||*ID*_B_*||*K*_*A*_||*X*_*A*_).
  *S* checks whether *Z*_*AS*_ and *Z*_AS_* are same. If *Z*_*AS*_ ≠ *Z*_AS_*, *S* aborts the process. *S* also computes
  *K*_*BS*_ = *T*_*s*_(*K*_*B*_) = *T*_*sb*_(*x*) *mod p*, {*ID*_*B*_, *Z*_BS_*, *Z*_*BA*_} = *D*_*KBS*_ (*M*_*BS*_), *X*_*B*_ = *H*(*ID*_*B*_||*s*), *Z*_*BS*_ = *H*(*ID*_*B*_||*K*_*B*_||*K*_*A*_||*X*_*B*_).
  *S* checks whether *Z*_*BS*_ and *Z*_BS_* are same. If *Z*_*BS*_ ≠ *Z*_BS_*, *S* aborts the process. *S* also checks whether *ID*_B_* of *A*’s message and *ID*_*B*_ of *B*’s message are same. If not, *S* aborts the process.
  After that, *S* computes
  *Z*_*SA*_ = *H*(*ID*_*A*_||*ID*_*B*_||*K*_*A*_||*K*_*B*_||*X*_*A*_), *Z*_*SB*_ = *H*(*ID*_*B*_||*ID*_*A*_||*K*_*B*_||K_A_||*X*_*B*_), *M*_*SA*_ = *E*_*KAS*_(*ID*_*B*_, *K*_*B*_, *Z*_*SA*_, *Z*_*BA*_), *M*_*SB*_ = *E*_*KBS*_(*ID*_*A*_, *K*_*A*_, *Z*_*SB*_).
  *S* sends *M*_3_ = {*M*_*SA*_, *M*_*SB*_} to *A*.
4. After receiving {*M*_*SA*_, *M*_*SB*_} from *S*, *A* computes
  {*ID*_*B*_, *K*_*B*_, *Z*_BA_*, *Z*_SA_*} = *D*_*KAS*_ (*M*_*SA*_), *Z*_*SA*_ = *H*(*ID*_*A*_||*ID*_*B*_||*K*_*A*_||*K*_*B*_||*X*_*A*_).
  If *Z*_*SA*_ ≠ *Z*_SA_*, *A* aborts the process. *A* also computes
  *K*_*AB*_ = *T*_*a*_(*K*_*B*_) = *T*_*ab*_(*x*) *mod p*, *Z*_*BA*_ = *H*(*ID*_*B*_||*K*_*AB*_).
  If *Z*_*BA*_ ≠ *Z*_BA_*, *A* aborts the process, otherwise *A* sets *K*_*AB*_ as a session key. *A* also computes
  *Z*_*AB*_ = *H*(*ID*_*A*_||*ID*_*B*_||*K*_*AB*_).
  *A* sends *M*_5_ = {*M*_*SB*_, *Z*_*AB*_} to *B*.
5. After receiving {*M*_*SB*_, *Z*_AB_*} from *A*, *B* computes
  {*ID*_*A*_, *K*_*A*_, *Z*_SB_*} = *D*_*KBS*_ (*M*_*SB*_), *Z*_*SB*_ = *H*(*ID*_*B*_||*ID*_*A*_||*K*_*B*_||*K*_*A*_||*X*_*B*_).
  If *Z*_*SB*_ ≠ *Z*_SB_*, *B* aborts the process. *B* also computes
  *Z*_*AB*_ = *H*(*ID*_*A*_||*ID*_*B*_||*K*_*AB*_).
  If *Z*_*AB*_ ≠ *Z*_AB_*, *B* aborts the process. Otherwise *B* sets *K*_*AB*_ as a session key.

### 4.4 Password change phase

User *A* connects his smart card *SC*_*A*_ to the terminal and inputs his identifier *A*, password and biometrics *bm*_*A*_. *SC*_*A*_ computes *X*_*A*_ = *G*_*A*_ ⊕ *H*(*ID*_*A*_||*pw*_*A*_||*h*(*bm*_*A*_)) and *F*_A_^*^ = *H*(*ID*_*A*_||*pw*_*A*_||*h*(*bm*_*A*_)||*X*_*A*_), and checks whether *F*_*A*_ and *F*_A_^*^ are same. If *F*_*A*_ ≠ *F*_A_^*^, *SC*_*A*_ aborts the process. Otherwise *SC*_*A*_ requests the user to input a new password *newpw*_*A*_. *SC*_*A*_ computes *G*_*A*_^*new*^ = *H*(*ID*_*A*_||*newpw*_*A*_||*h*(*bm*_*A*_)) ⊕ *X*_*A*_ and *F*_*A*_^*new*^ = *H*(*ID*_*A*_||*newpw*_*A*_||*h*(*bm*_*A*_)||*X*_*A*_), and replaces <*G*_*A*_, *F*_*A*_> of his memory with <*G*_*A*_^*new*^, *F*_*A*_^*new*^>.

## 5. Security analysis of the proposed scheme

In this section, we analyse the security properties of the proposed scheme. First, we prove the correctness of the session key between users by using BAN logic [59]. Next, we simulate the proposed scheme for the formal security analysis by using AVISPA(Automated validation of internet security protocol and application) tool [60]. Last, we demonstrate the proposed scheme can resist various kinds of attacks.

### 5.1 Authentication proof based on BAN logic

#### Notations and Rules

We define *P* and *Q* as the specific participators, *S* is the trusted server, and *X* is the formula (statement). Some notations and rules of BAN logic are as follows [59].

*P* |≡ *X*: *P* believes *X*.
*P*⊲*X*: *P* sees *X*.
*P* |∼ *X*: *P* once said *X*.
*P* |⇒ *X*: *P* has jurisdiction over *X*.
#(*X*): *X* is fresh.
$P\overset{K}{↔}Q$: *K* is a shared secret key between *P* and *Q*.
{*X*}_*K*_: Formula *X* are encrypted under the key *K*.
<*X*>_*Y*_: *X* combined with the formula *Y*.
$R_{1}:\frac{P|\equiv Q\overset{K}{↔}P,P⊲{\left\{X\right\}}_{K}}{P|\equiv Q|∼X}$ (Message-meaning rule): if *P* believes that the key *K* is shared with *Q* and receives a message containing *X* encrypted under *K*, then *P* believes that *Q* once said *X*.
$R_{2}:\frac{P|\equiv \#(X),P|\equiv Q|∼X}{P|\equiv Q|\equiv X}$ (Nonce-verification rule): if *P* believes *X* is fresh and *Q* once said *X*, *P* believes *Q* believes *X*.
$R_{3}:\frac{P|\equiv Q|\Rightarrow X,P|\equiv Q|\equiv X}{P|\equiv Q|\equiv X}$ (Jurisdiction rule): if *P* believes that *Q* had jurisdiction right to *X* and believes *Q* believes *X*, *P* believes *X*.
$R_{4}:\frac{P|\equiv \#(X)}{P|\equiv \#(X,Y)}$ (Freshness rule): If *X* is a part of message(*X*, *Y*) and *X* is fresh, message (*X*, *Y*) is also fresh.
$R_{5}:\frac{P|\equiv Q|\equiv (X,Y)}{P|\equiv Q|\equiv X}$ (Belief rule 1): If *P* believes *Q* believes the message set (*X*, *Y*), *P* also believes *Q* believes the message *X*.
$R_{6}:\frac{P|\equiv X,P|\equiv Y}{P|\equiv (X,Y)}$ (Belief rule 2): If *P* believes the message *X* and *Y*, *P* also believes the message set (*X*, *Y*).
$R_{7}:\frac{P|\equiv Q\overset{K}{↔}P,P⊲{\left\{X\right\}}_{K}}{P⊲X}$ (See rule): if *P* believes that the key *K* is shared with *Q* and receives a message containing *X* encrypted under *K*, then *P* sees *X*.

#### Goals

The session key exchange protocol should achieve the following goals:

$$Goal_{1}:A|\equiv A\overset{K_{AB}}{↔}B$$

$$Goal_{2}:B|\equiv A\overset{K_{AB}}{↔}B$$

$$Goal_{3}:A|\equiv B|\equiv A\overset{K_{AB}}{↔}B$$

$$Goal_{4}:B|\equiv A|\equiv A\overset{K_{AB}}{↔}B$$

#### Idealize

We idealize the communication messages of the proposed scheme as follows:

$$M_{1}:A\to S:{\left\{ID_{A},ID_{B},Z_{AS}\right\}}_{H(ID_{A}\|s)},T_{a}(x)$$

$$M_{2}:B\to S:{\left\{ID_{B},Z_{BS},Z_{BA}\right\}}_{H(ID_{B}\|s)},T_{b}(x)$$

$$M_{3}:S\to A:{\left\{ID_{B},T_{b}(x),Z_{SA},Z_{BA}\right\}}_{H(ID_{A}\|s)}$$

$$M_{4}:S\to A:\{{\left(ID_{B},A\overset{K_{AB}}{↔}B\right)}_{A\overset{K_{AB}}{↔}B}{\}}_{H(ID_{A}\|s)}$$

$$M_{5}:S\to B:{\left\{ID_{A},T_{a}(x),Z_{SB}\right\}}_{H(ID_{B}\|s)}$$

$$M_{6}:A\to B:{\left(ID_{A},ID_{B},A\overset{K_{AB}}{↔}B\right)}_{A\overset{K_{AB}}{↔}B}$$

#### Assumptions

The initial assumptions of the proposed scheme are as follows:

$$A_{1}:A|\equiv a$$

$$A_{2}:A|\equiv \#(a)$$

$$A_{3}:B|\equiv b$$

$$A_{4}:B|\equiv \#(b)$$

$$A_{5}:A|\equiv A\overset{H(ID_{A}\|s)}{↔}S$$

$$A_{6}:B|\equiv B\overset{H(ID_{B}\|s)}{↔}S$$

$$A_{7}:A|\equiv S|\Rightarrow T_{b}(x)$$

$$A_{8}:B|\equiv S|\Rightarrow T_{a}(x)$$

#### Analysis

According to *M*_3_ and *A*_5_, we apply the message meaning rule (*R*_1_) and the See rule (*R*_7_), we can obtain:

$$S_{1}:\frac{A|\equiv A\overset{H(ID_{A}\|s)}{↔}S,A⊲{\left\{ID_{B},T_{b}(x),Z_{SA},Z_{BA}\right\}}_{H(ID_{A}\|s)}}{A|\equiv S|∼\{ID_{B},T_{b}(x),Z_{SA},Z_{BA}\},A⊲Z_{BA}}$$

According to *Z*_*SA*_ = *H*(*ID*_*A*_||*ID*_*B*_||*T*_*a*_(*x*)||*T*_*b*_(*x*)||*X*_*A*_), *A*_2_ and *M*_3_, we apply the Freshness rule (*R*_4_), we can obtain:

$$S_{2}:\frac{A|\equiv \#(a)}{A|\equiv \#(Z_{SA})},\frac{A|\equiv \#(Z_{SA})}{A|\equiv \#(ID_{B},T_{b}(x),Z_{SA},Z_{BA})}$$

According to *S*_1_ and *S*_2_, we apply the Nonce-verification rule (*R*_2_) and Belief rule 1(*R*_5_), we can obtain:

$$\frac{A|\equiv \#(ID_{B},T_{b}(x),Z_{SA}),A|\equiv S|∼\{ID_{B},T_{b}(x),Z_{SA},Z_{BA}\}}{A|\equiv S|\equiv (ID_{B},T_{b}(x),Z_{SA},Z_{BA})}$$

$$S_{3}:\frac{A|\equiv S|\equiv (ID_{B},T_{b}(x),Z_{SA},Z_{BA})}{A|\equiv S|\equiv T_{b}(x)}$$

According to *S*_3_ and *A*_7_, we apply the Jurisdiction rule (*R*_3_), we can obtain:

$$S_{4}:\frac{A|\equiv S|\Rightarrow T_{b}(x),A|\equiv S|\equiv T_{b}(x)}{A|\equiv T_{b}(x)}$$

According to *S*_4_, *A*_1_ and *K*_*AB*_ = *T*_*a*_(*T*_*b*_(x)) = (*a*, *T*_*b*_(*x*)), we apply the Belief rule 2(*R*_6_), we can obtain:

$$S_{5}:\frac{A|\equiv a,A|\equiv T_{b}(x)}{A|\equiv A\overset{K_{AB}}{↔}B}:(Goal_{1})$$

According to *M*_5_ and *A*_6_, we apply the message meaning rule (*R*_1_), we can obtain:

$$S_{6}:\frac{B|\equiv B\overset{H(ID_{B}\|s)}{↔}S,B⊲{\left\{ID_{A},T_{a}(x),Z_{SB}\right\}}_{H(ID_{B}\|s)}}{B|\equiv S|∼\{ID_{A},T_{a}(x),Z_{SB}\}}$$

According to *Z*_*SB*_ = *H*(*ID*_*B*_||*ID*_*A*_||*T*_*b*_(*x*)||*T*_*a*_(*x*)||*X*_*B*_), *A*_4_ and *M*_5_, we apply the Freshness rule(*R*_4_), we can obtain:

$$S_{7}:\frac{B|\equiv \#(b)}{B|\equiv \#(Z_{SB})},\frac{B|\equiv \#(Z_{SB})}{B|\equiv \#(ID_{A},T_{a}(x),Z_{SB})}$$

According to *S*_6_ and *S*_7_, we apply the Nonce-verification rule (*R*_2_) and the Belief rule 1(*R*_5_), we can obtain:

$$\frac{B|\equiv \#(ID_{A},T_{a}(x),Z_{SB}),B|\equiv S|∼\{ID_{A},T_{a}(x),Z_{SB}\}}{B|\equiv S|\equiv (ID_{A},T_{a}(x),Z_{SB})}$$

$$S_{8}:\frac{B|\equiv S|\equiv (ID_{A},T_{a}(x),Z_{SB})}{B|\equiv S|\equiv T_{a}(x)}$$

According to *S*_8_ and *A*_8_, we apply the Jurisdiction rule (*R*_3_), we can obtain:

$$S_{9}:\frac{B|\equiv S|\Rightarrow T_{a}(x),B|\equiv S|\equiv T_{a}(x)}{B|\equiv T_{a}(x)}$$

According to *S*_9_, *A*_3_ and *K*_*AB*_ = *T*_*b*_(*T*_*a*_(x)) = (*b*, *T*_*a*_(*x*)), we apply the Belief rule 2 (*R*_6_), we can obtain:

$$S_{10}:\frac{B|\equiv b,B|\equiv T_{a}(x)}{B|\equiv A\overset{K_{AB}}{↔}B}:(Goal_{2})$$

According to *M*_4_, *S*_1_ and *S*_5_, we apply the message meaning rule (*R*_1_), we can obtain:

$$S_{11}:\frac{A|\equiv A\overset{K_{AB}}{↔}B,A⊲(ID_{B},A\overset{K_{AB}}{↔}B{)}_{A\overset{K_{AB}}{↔}B}}{A|\equiv B|∼\{ID_{B},A\overset{K_{AB}}{↔}B\}}$$

According to *A*_2_ and *K*_*AB*_ = *T*_*b*_(*T*_*a*_(x)) = (*a*, *T*_*b*_(*x*)), we apply the Freshness rule (*R*_4_), we can obtain:

$$S_{12}:\frac{A|\equiv \#(a)}{A|\equiv \#(A\overset{K_{AB}}{↔}B)}$$

According to *S*_11_ and *S*_12_, we apply the Nonce-verification rule (*R*_2_), we can obtain:

$$S_{13}:\frac{A|\equiv \#(A\overset{K_{AB}}{↔}B),A|\equiv B|∼\{A\overset{K_{AB}}{↔}B\}}{A|\equiv B|\equiv (A\overset{K_{AB}}{↔}B)}:(Goal_{3})$$

According to *M*_6_ and *S*_10_, we apply the message meaning rule (*R*_1_), we can obtain:

$$S_{14}:\frac{B|\equiv A\overset{K_{AB}}{↔}B,B⊲{\left(ID_{A},A\overset{K_{AB}}{↔}B\right)}_{A\overset{K_{AB}}{↔}B}}{B|\equiv A|∼\{ID_{A},A\overset{K_{AB}}{↔}B\}}$$

According to *A*_4_ and *K*_*AB*_ = *T*_*a*_(*T*_*b*_(x)) = (*b*, *T*_*a*_(*x*)), we apply the Freshness rule(*R*_4_), we can obtain:

$$S_{15}:\frac{B|\equiv \#(b)}{B|\equiv \#(A\overset{K_{AB}}{↔}B)}$$

According to *S*_14_ and *S*_15_, we apply the Nonce-verification rule (*R*_2_), we can obtain:

$$S_{16}:\frac{B|\equiv \#(A\overset{K_{AB}}{↔}B),B|\equiv A|∼\{A\overset{K_{AB}}{↔}B\}}{B|\equiv A|\equiv (A\overset{K_{AB}}{↔}B)}:(Goal_{4})$$

### 5.2 Validation test based on AVISPA

In this section, we simulate the proposed scheme for the formal security analysis using AVISPA, which is widely used to verify the security properties of designed protocol such as resistance against replay attack and man-in-the-middle attack. This tool implements four back-ends: On-the-Fly-Model-Check(OFMC), Constraint Logic based Attack Searcher(CL-AtSe), SAT-based Model-Checker(SATMC) and Three Automata based on Automatic Approximations for the Analysis of Security Protocols(TA4SP), which are given in details in [60]. In order to verify the security properties of the protocol using AVISPA, it needs to be specified in HLPSL(High Level Protocol Specification Language), which is a role-based languages: basic roles for representing each participant role, and composition roles for representing scenarios of basic roles. Each role is independent from the other, communicating with the other roles by channels [60]. The output format is generated by using one of the four back-ends.

#### Specifying the proposed protocol

**Fig 3:**
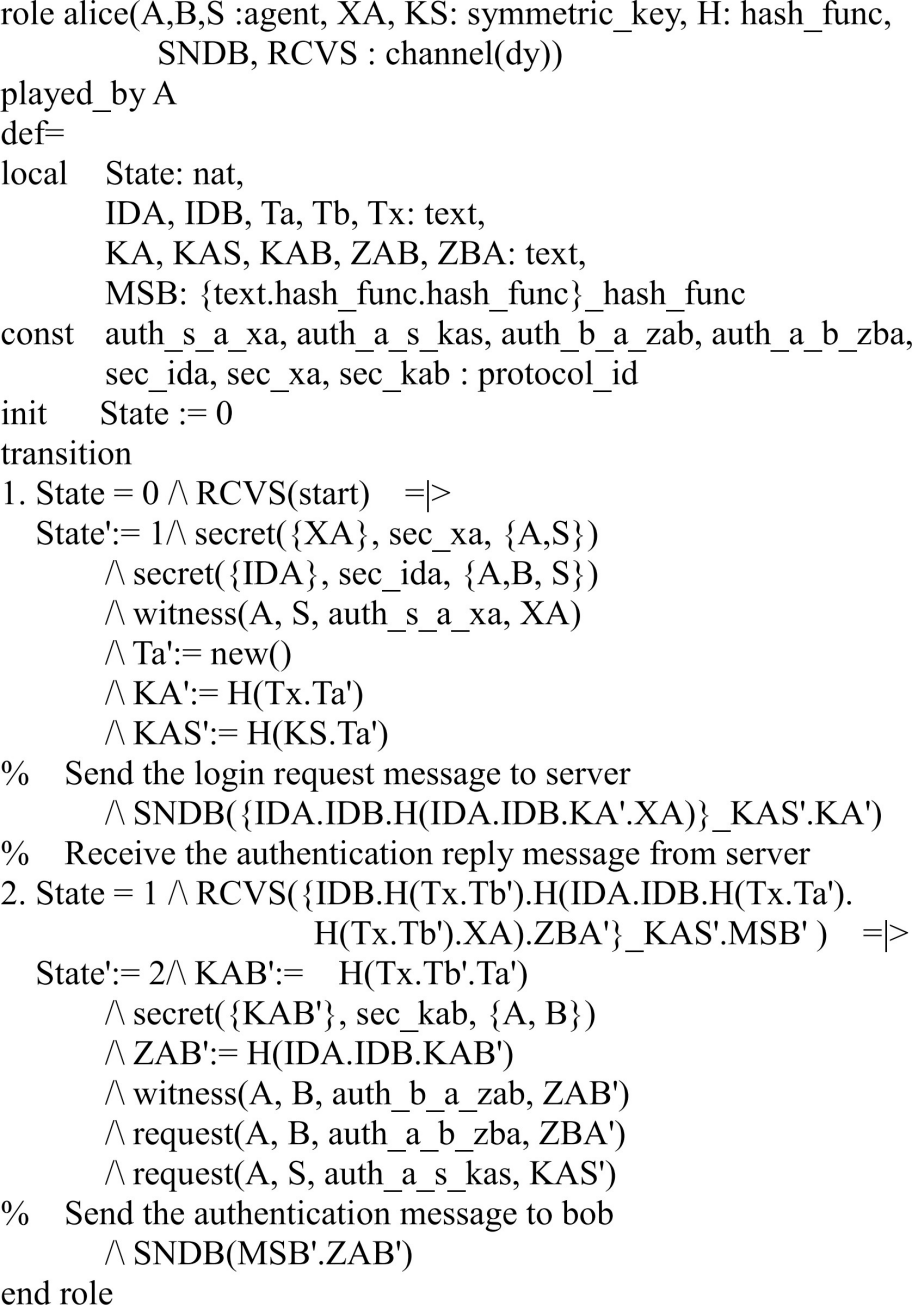
Role specification in HLPSL for the user *A*.

**Fig 4:**
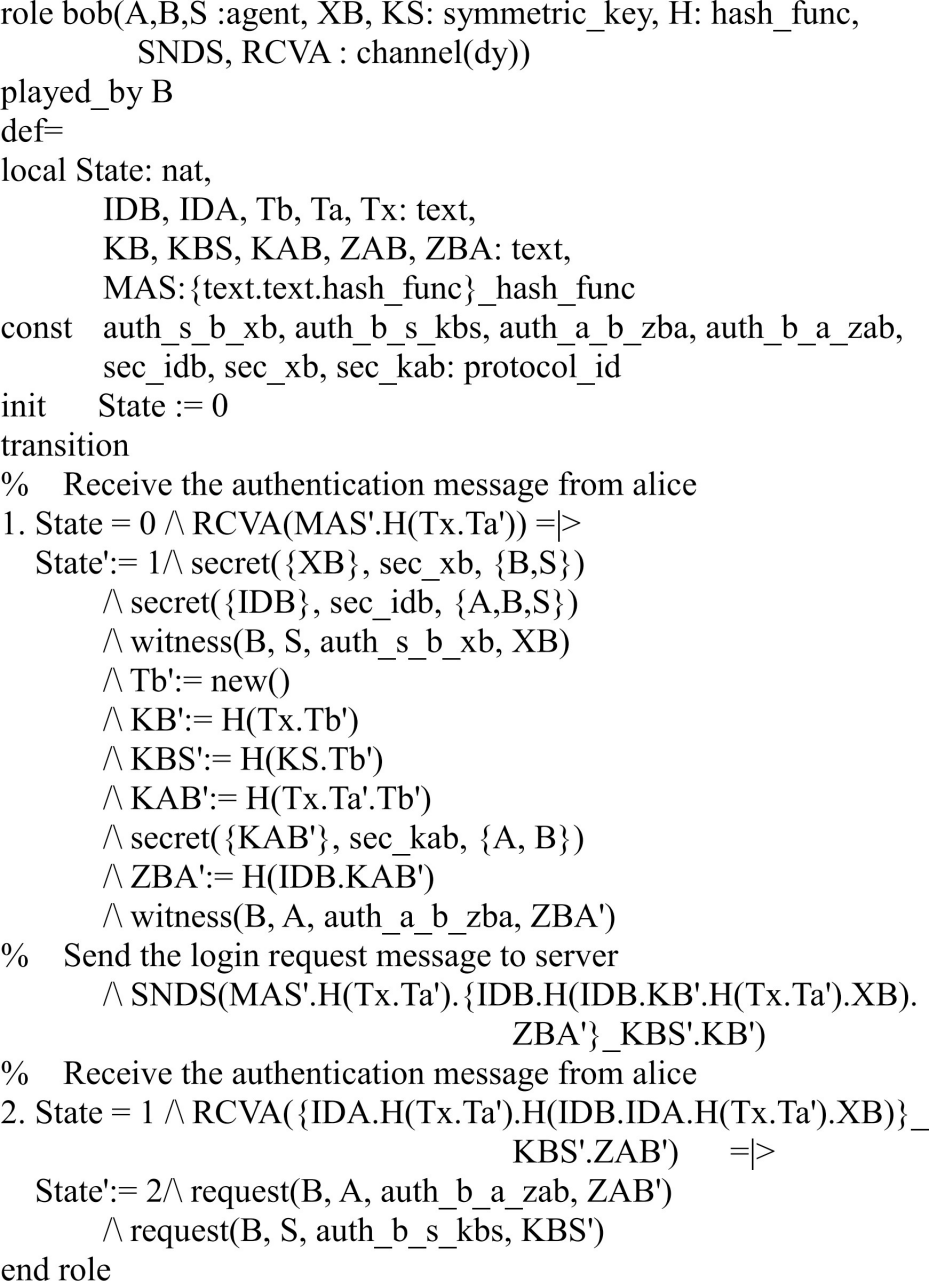
Role specification in HLPSL for the user *B*.

**Fig 5:**
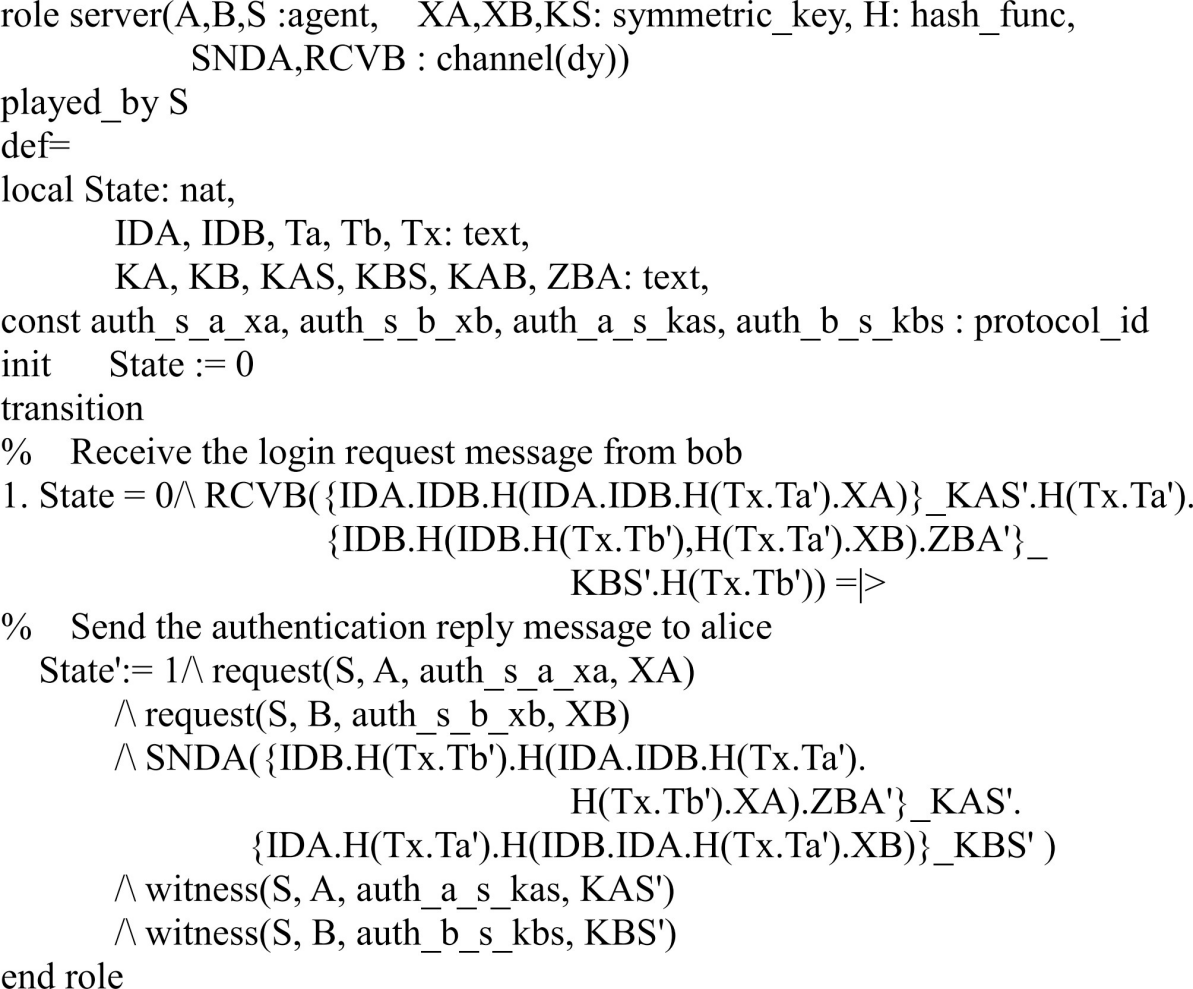
Role specification in HLPSL for the server *S*.

In our HLPSL implementation, we define three basic roles for users *A*, *B*, and server *S*. Figs 3, 4 and 5 shows the specifications in HLPSL for the role of users *A*, *B*, and server *S*.

**Fig 6:**
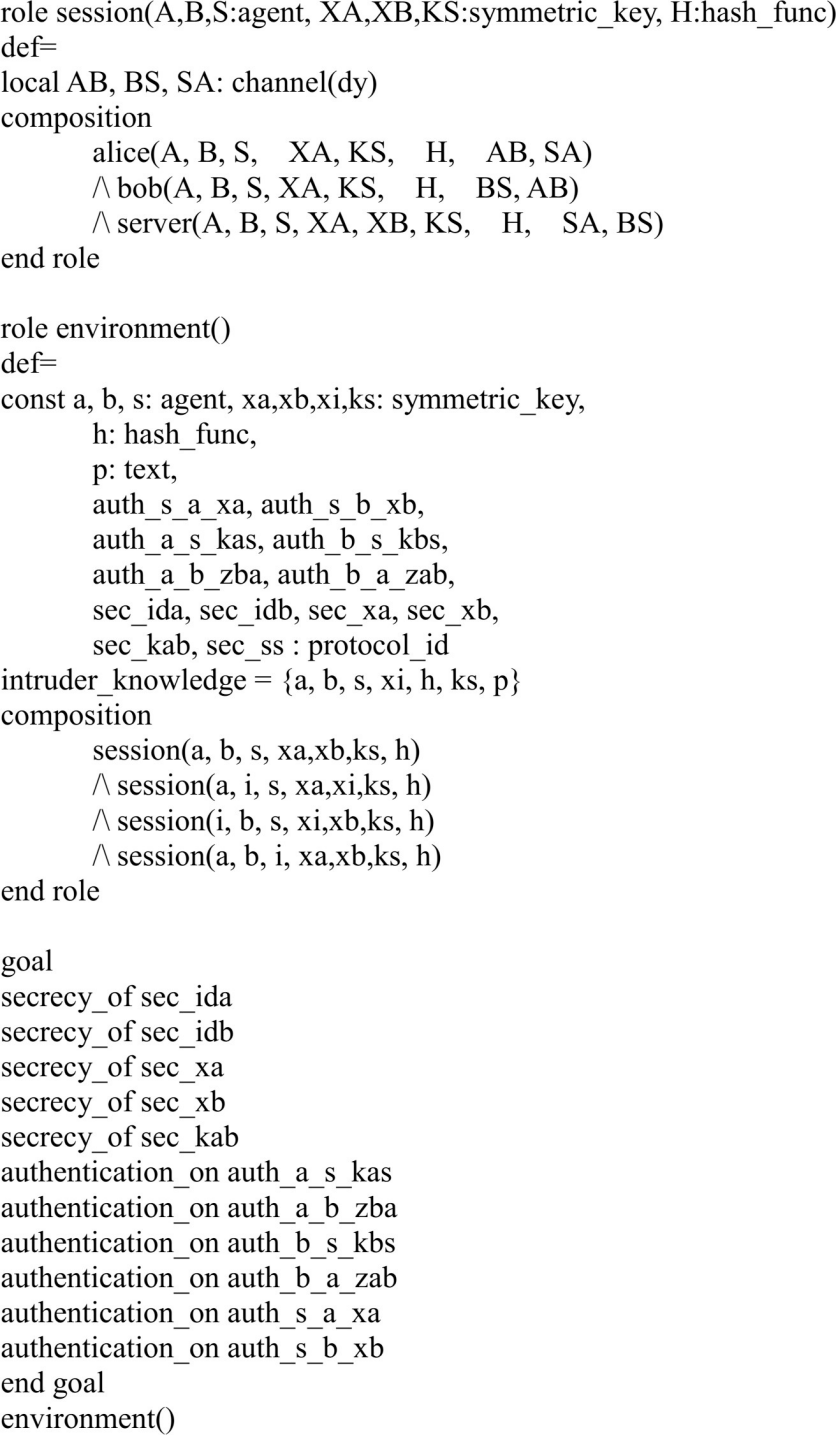
Role specification in HLPSL for the session, environment and goal.

In Fig 6, we shows the HLPSL implementation for the role of the session, environment and goal.

In our implementation, we verified the following five secrecy goals and six authentication properties.

- secrecy_of sec_ida: It represents that user *A*'s identifier *ID*_*A*_ is kept secret to the user *A*, *B* and server *S* only.
- secrecy_of sec_idb: It represents that user *B*'s identifier *ID*_*B*_ is kept secret to the user *A*, *B* and server *S* only.
- secrecy_of sec_xa: It represents that user *A*'s secret key *X*_*A*_ is kept secret to the user *A* and server *S* only.
- secrecy_of sec_xb: It represents that user *B*'s secret key *X*_*B*_ is kept secret to the user *B* and server *S* only.
- secrecy_of sec_kab: It represents that session key *K*_*AB*_ is kept secret to the user *A* and *B* only.
- authentication_on auth_a_s_kas: When user *A* receives the messages from server *S* and decrypts the message with *K*_*AS*_, *A* authenticates *S* based on *K*_*AS*_.
- authentication_on auth_a_b_zba: When user *A* receives *Z*_*BA*_ from the messages from *B*, *A* authenticates *B* based on *Z*_*BA*_.
- authentication_on auth_b_s_kbs: When user *B* receives the messages from server *S* and decrypts the message with *K*_*BS*_, *B* authenticates *S* based on *K*_*BS*_.
- authentication_on auth_b_a_zab: When user *B* receives *Z*_*AB*_ from the messages from *A*, *B* authenticates *A* based on *Z*_*AB*_.
- authentication_on auth_s_a_xa: When server *S* receives *X*_*A*_ from the messages from *A*, *S* authenticates *A* based on *X*_*A*_.
- authentication_on auth_s_b_xb: When server *S* receives *X*_*B*_ from the messages from *B*, *S* authenticates *B* based on *X*_*B*_.

#### Analysis of the results

**Fig 7:**
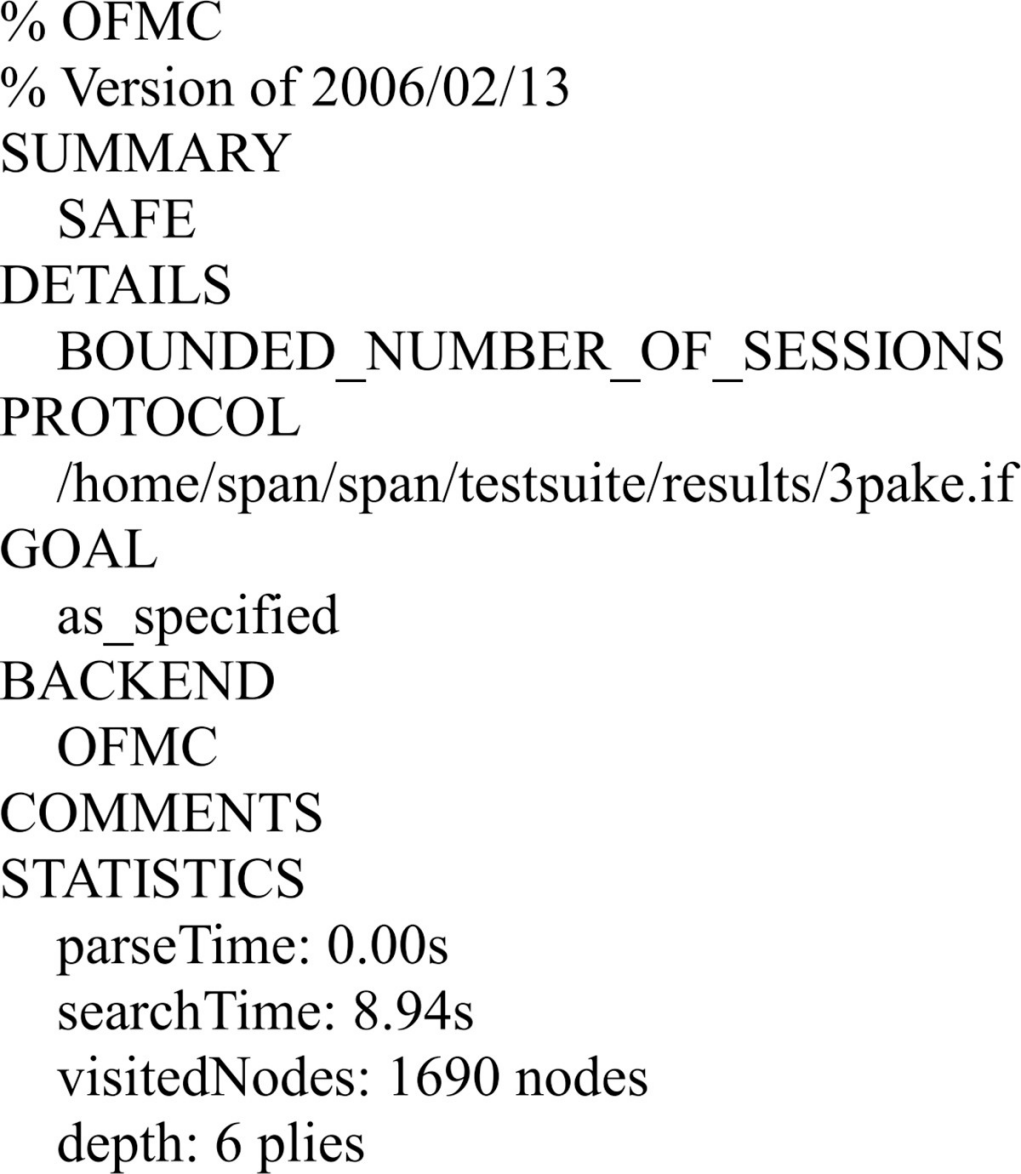
The result of the analysis using OFMC back-end.

**Fig 8:**
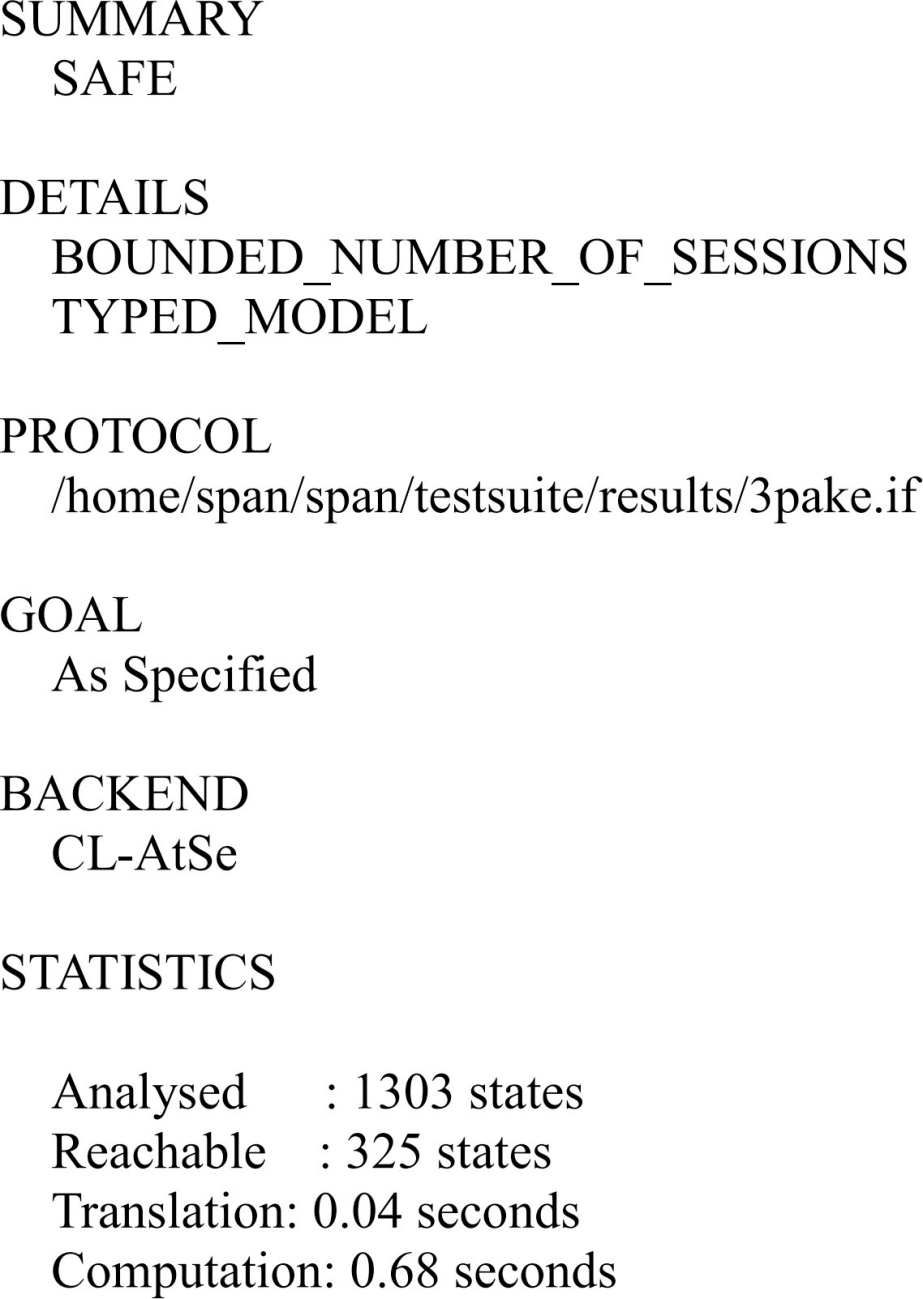
The result of the analysis using CL-AtSe back-end.

We have simulated the proposed scheme using FMC and CL-AtSe back-ends of AVISPA. The simulation results for the security verification is shown in Figs 7 and 8.

The results ensure that the proposed scheme is secure under the test of AVISPA using OFMC and CL-AtSe back-ends, and guarantees user anonymity, and it is also secure against the passive attacks and the active attacks, such as the replay attack and man-in-the-middle attack.

### 5.3 Informal security analysis

In this part, we demonstrate the proposed scheme can resist various kinds of attacks.

#### User anonymity

The proposed scheme provides user anonymity for key exchange. All message (*M*_*AS*_, *M*_*BS*_, *M*_*SA*_ and *M*_*SB*_) associated with the user’s identifier is encrypted with the shared secret key *K*_*XS*_ between the server *S* and the user *X*. The shared secret key *K*_*AS*_ is calculated from the random number *a* of the user *A* and the secret key *s* of the server *S* as follows: *K*_*AS*_ = *T*_*a*_(*T*_*s*_(*x*)) = *T*_*s*_(*T*_*a*_(*x*)).

Even if *T*_*a*_(*x*) and *T*_*s*_(*x*) is exposed, it is impossible to calculate *K*_*AS*_ or *a*, *s* according to *CDLP* and *CDHP* assumptions. Therefore, a third party cannot know the user’s identifier except user and server.

#### Off-line password guessing attack

The proposed scheme resists the password guessing attack. The proposed scheme does not use passwords during the authentication process but only uses passwords when accessing the smart card. The information registered on the user *A*’s smart card is {*G*_*A*_, *F*_*A*_, *p*, *x*, *K*_*S*_, *R*_*S*_, *H*(∙), *E*_*K*_(∙), *D*_*K*_(∙)}, and the information that can be used for guessing password is *G*_*A*_ = *H*(*ID*_*A*_||*pw*_*A*_||*h*(*bm*_*A*_))⊕*X*_*A*_ and *F*_*A*_ = *H*(*ID*_*A*_||*pw*_*A*_||*h*(*bm*_*A*_) ||*X*_*A*_). Suppose that an attacker steals user *A*’s smart card *SC*_*A*_ and knows his identifier *ID*_*A*_. Then the attacker must compute *PW*_A_* = *H*(*ID*_*A*_||*pw*_A_*||*h*(*bm*_*A*_)), *X*_A_^*^ = *G*_*A*_ ⊕ *PW*_A_* and *F*_A_^*^ = *H*(*ID*_*A*_||*pw*_A_*||*h*(*bm*_*A*_)||*X*_A_^*^) by using *ID*_*A*_ and any password *pw*_A_* to compare *F*_A_^*^ and *F*_*A*_ stored in *SC*_*A*_. However, *PW*_A_* cannot be calculated without knowing *h*(*bm*_*A*_) which is related *A*’s biometrics. Therefore, the attacker cannot guess the user’s password.

#### Privileged insider attack

The proposed scheme is secure against the privileged-insider attack. In the registration phase of the proposed scheme, only the user’s identifier is transmitted to the server through a secure channel and the user’s password is not transmitted to the server. Therefore, the privilege insider of the server cannot know the user’s password. Therefore, the proposed scheme is secure against this attack.

#### Stolen verifier attack

The proposed scheme is secure against stolen verifier attack. In the proposed scheme, there is no user registration table to authenticate user in the server. Therefore, the proposed scheme is secure against stolen verifier attack.

#### User impersonate attack

The proposed scheme is secure against the user impersonate attack and the forgery attack.

In order to impersonate as user *A*, the attacker *C* changes *K*_*A*_ to *K*_*C*,_ and sends a message {*M*_AS_* (= *E*_*KCS*_(*ID*_*A*_, *ID*_*B*_, *Z*_AS_*)), *K*_*C*_} to the server. The server receiving the message from attacker *C* computes *K*_*SC*_ from *K*_*C*_ and decrypts *M*_AS_* using *K*_*SC*_ to obtain *ID*_*A*_, *ID*_*B*_ and *Z*_AS_*. Next, server computes *X*_*A*_ = *H*(*ID*_*A*_||*s*) and *Z*_*AS*_ = *H*(*ID*_*A*_||*ID*_*B*_||*K*_*A*_||*X*_*A*_), and compares it with *Z*_AS_*. Therefore, the attacker has to know *X*_*A*_ = *H*(*ID*_*A*_||*s*) or *s*.

However, since *s* is a secret key of the server and *X*_*A*_ is a secret data that only user *A* has, the attacker *C* cannot know it, and thus the impersonate attack is impossible. Also, even if an attacker attempts to impersonate as the user *B*, he does not know *X*_*B*_ or *s*, so he cannot achieve the attack as before.

#### Man-in-the-middle attack

As above, since an attacker *C* cannot know *X*_*A*_ = *H*(*ID*_*A*_||*s*), *X*_*B*_ = *H*(*ID*_*B*_||*s*) or *s*, so he cannot modify the sender’s message or cannot change *K*_*A*_ and *K*_*B*_, and cannot achieve the man-in-the-middle attack.

#### Replay attack

If an attacker *C* sends the previous message {*M*_AS_*, *T*_a_*(*x*)} of the user *A*, according to *CDLP* and *CDHP* assumptions, he cannot know a*, so he does not calculate *Z*_*AB*_ in the fourth message of the proposed scheme.

If an attacker *C* sends the previous message {*M*_BS_*, *T*_b_*(*x*)} of the user *B*, *Z*_BS_* is calculated as *Z*_BS_* = *H*(*ID*_*B*_||*R*_A_*||*R*_B_*||*X*_*B*_). Since *Z*_*BS*_ is related to *R*_*A*_ and the server verifies the correctness of *Z*_*BS*_, it is impossible for the attacker *C* to achieve the replay attack.

#### Perfect forward security of session key

In the proposed scheme, the session key *K*_*AB*_ is calculated as *K*_*AB*_ = *T*_*a*_(*K*_*B*_) = *T*_*ab*_(*x*) *mod p*. It contains the random numbers *a* and *b* that are generated for each session.

Therefore, the proposed scheme provides the perfect forward secrecy of session key.

#### Known key security

In the proposed scheme, the session key *K*_*AB*_ is calculated as *K*_*AB*_ = *T*_*a*_(*K*_*B*_) = *T*_*ab*_(*x*) *mod p*. It contains the random numbers *a* and *b* that are generated for each session. Even if an attacker knows previous session key, he cannot calculate a new session key.

## 6. Performance comparisons

This section compares the computational cost and security performance of the proposed scheme with the recent similar 3PAKE techniques [23, 31, 38, 49, 50], of which three [23, 31, 38] attempted to provide user anonymity and others [49, 50] use smart card. The notations used for comparison of computational cost are as follows.

t_c_: time needed for Chebyshev polynomial operation
t_e_: time needed for a scalar multiplication on elliptic curve
t_s_: time needed for symmetric encryption/decryption operation
t_m_: time needed for a modular squaring operation
t_q_: time needed for a square root modulo N operation
t_h_: time needed for one-way hash function operation

Table 2 shows the comparison of the computational cost of the six schemes, including the proposed scheme.

**Table 2:** Comparison of the computational cost between the proposed scheme and other 3PAKE scheme.

	Xie et al.[23]	Lu et al.[31]	Li et al.[38]	Amin et al.[39]	Islam et al.[41]	proposed
A	3te + 2ts + 4th	3tc + 4ts + 4th	4tc + 1tm + 5th	8th	2tc + 4ts + 2th	3tc + 2ts + 6th
B	3te + 2ts + 5th	2tc + 3ts + 5th	4tc + 1tm + 5th	9th	2tc + 4ts + 2th	3tc + 2ts + 6th
S	2te + 4ts + 7th	5tc + 5ts+ 7th	4tc + 2tq + 5th	10th	4ts + 3th	2tc + 4ts + 6th
Total	8te + 8ts+16th	10tc + 12ts+ 16th	12tc+2tm+2tq+15th	27th	4tc + 12ts + 7th	8tc + 8ts + 18th
Round	4	5	6	4	4	4
Messages	5	7	6	6	8	4

Table 3 shows the comparative evaluation of the security function between the proposed scheme and other 3PAKE schemes.

**Table 3:** Comparative evaluation of the security function between the proposed scheme and other 3PAKE schemes.

	Xie et al.[23]	Lu et al.[31]	Li et al.[38]	Amin et al.[39]	Islam et al.[41]	proposed
Provision of User anonymity	Yes	No	Yes	No	No	Yes
Protection of Privileged insider attack	No	Yes	Yes	No	Yes	Yes
Protection of password guessing attack	Yes	Yes	Yes	Yes	Yes	Yes
Protection of User impersonate attack	Yes	Yes	Yes	Yes	Yes	Yes
Provision of Password change phase	No	Yes	Yes	Yes	Yes	Yes
Secrecy of Password change phase	-	No	Yes	Yes	Yes	Yes
Password change without server’s help	-	No	No	Yes	Yes	Yes
Without timestamp	Yes	Yes	Yes	Yes	No	Yes
Using smart card	No	No	No	Yes	Yes	Yes

As shown in Table 2 and Table 3, the proposed scheme outperforms the other schemes in terms of the security functions presented. Xie’s scheme provides user anonymity, but his scheme is vulnerable to the privileged insider attack. Lu et al.’s scheme attempted to provide user anonymity, but did not achieve it. There are weaknesses at the session key establishment phase and the password change phase of his scheme. Li’s scheme provides user anonymity, but in his scheme there are more rounds, messages and computational cost than our proposed scheme. Amin’s and Islam’s scheme are superior to our proposed scheme in terms of computational cost, but do not provide user anonymity for key exchange.

## 7. Conclusion

In this paper, we analyse the Lu et al.’s scheme and point out its weakness, and propose a round-effective 3PAKE protocol based on chaotic maps using smart card to provide with user anonymity. In the proposed scheme, there is no information related to the user’s password at the server side and users share the secret key with the server, which is derived by the server’s secret key and his identifier. The proposed scheme is more efficient than other schemes in terms of number of rounds and computational cost, and it is formally analysed based on BAN logic and AVISPA tool, and can protect against various attacks as shown through informal security analysis. The proposed scheme is suitable for authentication and key agreement in a wireless network environment.
